# 

**DOI:** 10.1192/bjb.2025.10177

**Published:** 2026-06

**Authors:** Ratan Sarkar

**Affiliations:** Department of Education, Tezpur University (a Central University), Assam, India.

**Figure:**
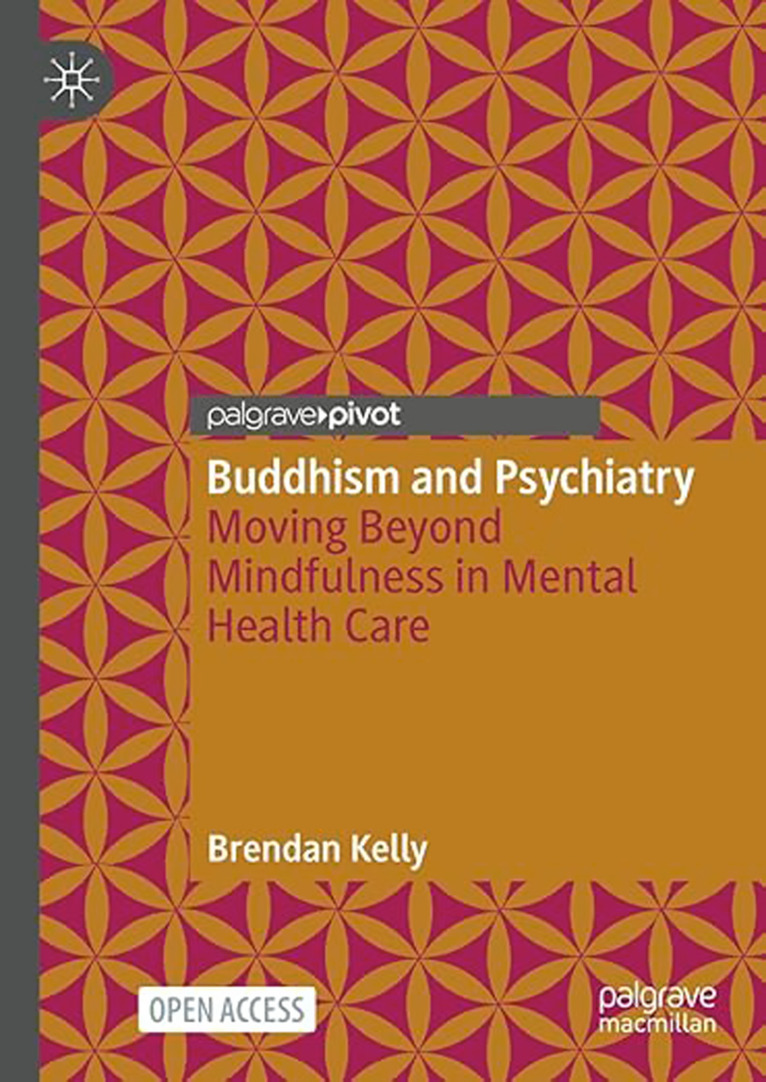

Brendan Kelly’s *Buddhism and Psychiatry* is a bold and timely book that situates the contemporary fascination with mindfulness within a broader and more textured dialogue between Buddhist thought and psychiatric practice. Kelly begins with the ‘rise and rise of mindfulness’ and its diffusion into healthcare, education and everyday culture (p. 3). While he acknowledges its therapeutic usefulness, he also cautions against its oversimplification: ‘Mindfulness is not passive; it is active. It means tuning in, not dropping out’ (p. 4). This insight sets the stage for a book that insists mindfulness is but one thread in the complex tapestry linking Buddhism and psychiatry.

The book’s central aim is to explore how Buddhist philosophy – particularly ideas of *dukkha* (unsatisfactoriness), impermanence (*anicca*) and non-self (*anattā*) – can enrich psychiatric understandings of suffering. In doing so, Kelly fulfils his promise to move beyond mindfulness as a commodified wellness tool and instead to recover its ethical and philosophical roots. The chapter on mental disorder offers a striking comparison between Buddhist conceptions of suffering and psychiatric frameworks such as DSM-5-TR and ICD-11. Kelly reminds us that ‘diagnosis is a very human process’ (p. 10) that should be aimed at understanding rather than categorising. By placing Buddhist insights alongside psychiatric criteria, he calls for humility, flexibility and compassion in clinical practice.

One of the book’s most engaging sections is the discussion of equanimity (*upekkhā*), framed as both a Buddhist ‘immeasurable’ and a quality vital for contemporary mental healthcare (pp. 69–74). Similarly, his treatment of Tibetan practices and the Abhidharma (pp. 51–4) demonstrates originality, extending the conversation well beyond the well-worn terrain of mindfulness-based interventions. Kelly writes with clarity, weaving clinical experience with scholarship in a style that is accessible to both specialists and general readers.

The book’s strengths lie in its scope, originality and readability. It offers a valuable corrective to psychiatry’s over-reliance on rigid diagnostic categories, encouraging instead a perspective grounded in impermanence and relationality. For psychiatrists, psychologists and allied professionals, the book provides both intellectual stimulation and practical guidance on integrating compassion into everyday care.

At the same time, limitations exist. The critique of mindfulness’s commodification, while present, might have benefited from deeper engagement with Buddhist voices from Asia, where questions of cultural appropriation and distortion are most keenly felt. Additionally, the breadth of themes occasionally results in brevity, leaving some discussions suggestive rather than fully developed.

Personally, reading this book has reshaped how I think about psychiatry’s role. The Buddhist reminder that ‘all will change’ (p. 21) urges me to treat diagnoses as provisional rather than permanent labels. More importantly, Kelly’s reflection that ‘we suffer together and so we heal together’ (p. 25) reinforces compassion as the ethical cornerstone of mental healthcare.

Ultimately, *Buddhism and Psychiatry* is a reflective and provocative invitation: to see psychiatry not only as a science of illness but as a shared human quest to alleviate suffering with wisdom, humility and care.

